# *MACROD2* deletions cause impaired PARP1 activity and chromosome instability in colorectal cancer

**DOI:** 10.18632/oncotarget.25887

**Published:** 2018-09-04

**Authors:** Anuratha Sakthianandeswaren, Marie J. Parsons, Dmitri Mouradov, Oliver M. Sieber

**Affiliations:** Oliver M. Sieber: Systems Biology and Personalised Medicine Division, The Walter and Eliza Hall Institute of Medical Research, Parkville, Victoria, Australia; Department of Medical Biology, The University of Melbourne, Parkville, Victoria, Australia; Department of Surgery, The University of Melbourne, Parkville, Victoria, Australia; Department of Biochemistry & Molecular Biology, Monash University, Clayton, Victoria, Australia

**Keywords:** MACROD2, colorectal cancer, ADP-ribosylation, PARP1, chromosome instability

The *MACRO domain containing 2* (*MACROD2*) locus on chromosome 20p12.1 is a frequent site of focal deletion in various human malignancies, including cancers of the large intestine (colorectal cancer, CRC), stomach, esophagus, cervix, uterus, lung, liver, and thyroid [1]. *MACROD2* encodes a mono-ADP-ribosylhydrolase involved in the regulation of protein ADP-ribosylation, a posttranslational modification implicated in multiple cellular processes such as chromatin reorganization, DNA damage response, transcriptional control, mitosis and apoptosis [2].

An important target of MACROD2 regulation is poly(ADP-ribose) polymerase 1 (PARP1), a principal sensor of single strand breaks (SSBs) and double strand breaks (DSBs) [3]. In response to DNA nicks or breaks, PARP1 binds to the site of damage and polymerises poly(ADP-ribose) PAR chains onto histones and other proteins, including itself, initiating a DNA damage response. The DNA damage signal established by PARP1 leads to the recruitment of repair factors and activation of effector proteins, comprising the key regulators ATM, ATR and DNA-dependent protein kinase (DNA-PK) [4]. Protein PARylation is a dynamic process which is reversed by poly(ADP-ribose) glycohydrolase (PARG) which cleaves the ribose-ribose bonds in the PAR chain. However, MACROD2 recruitment and enzymatic activity are required to remove the terminal autoinhibitory mono-ADP-ribose moiety from PARP1 and its subsequent reactivation [3]. MACROD2 phosphorylation by ATM has been shown to create a negative feedback loop resulting in MACROD2 nuclear export upon DNA damage, thereby limiting MACROD2 recruitment to DNA lesions and PARP1 reactivation [5].

Recently published work by our group has now fine-mapped the *MACROD2* deletion spectrum in human CRC, confirming a whole-gene or microdeletion prevalence of ∼30%, and revealed that *MACROD2* deletions cause impaired PARP1 activity and chromosome instability (CIN) in a gene-dosage dependent manner. In CRC cells, *MACROD2* loss resulted in increased levels of PARP1 mono-ADP-ribosylation and concomitant repression of PARP1 transferase activity, resulting in deficient DNA break repair and increased sensitivity to genotoxic stress induced DNA damage. Homologous recombination (HR) mediated DSB repair, a major DNA repair pathway mediated by PARP1, was shown to be defective. Similar observations have been reported for PARP1-deficient cells [6]. In-frame microdeletions in *MACROD2* affecting the macro domain were further shown to result in loss of protein recruitment to sites of DNA damage.

Critically, *MACROD2* loss was found to promote CIN, with a gene dosage-dependent increase in structural and numerical chromosomal abnormalities upon prolonged passage of CRC cells. The acquisition of CIN was accompanied by increased chromosome missegregation errors, including anaphase bridges, lagging chromosomes and micronuclei formation. Similar observations were made for *Macrod2* knockout mouse embryonic fibroblast (MEF) cells, and corresponding CIN phenotypes have been reported for *Parp1* knockout MEFs [6]. Accordingly, treatment of *MACROD2* wild-type CRC cells with the PARP1 inhibitor olaparib recapitulated the spectrum of chromosome abnormalities and missegregation errors observed with *MACROD2* deletion. In primary tumors, *MACROD2* deletion was associated with an increased number of chromosome segments and aneuploidy as estimated from single-nucleotide polymorphism (SNP) array data, independent of clinical features and other known drivers of CIN including *APC*, *TP53*, *FBXW7* and *BCL9L*. Notably, MACROD2-deficient CRC cells also displayed evidence of centrosome amplification which may additionally promote the acquisition of CIN, a phenotype which has also been identified in the context of PARP1 deficiency [7].

*In vivo* studies utilizing human CRC xenograft models and a mouse genetic model of CRC further support a role for *MACROD2* as a haploinsufficient tumor suppressor gene. *Macrod2*-knockout in CRC cells promoted xenograft growth, and *Apc*^*Min/+*^ mice with homozygous or heterozygous *Macrod2*-knockout exhibited enhanced intestinal tumorigenesis, with a significant increase in tumor number and volume. Consistent with our findings, Parp1-deficient mice examined for an alternative model of sporadic colon tumorigenesis (azoxymethane treatment) were previously found to display increased colon tumorigenesis [8].

**Figure 1:**
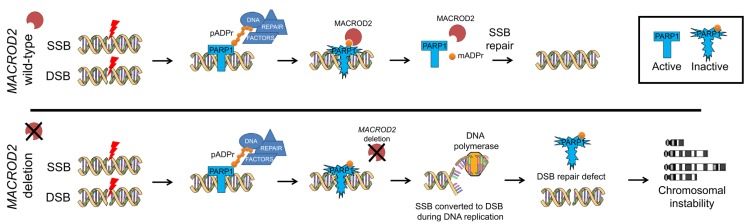
Focal deletion of *MACROD2* results in deficient MACROD2 mono-ADP-ribosylhydrolase activity As a consequence, removal of the terminal autoinhibitory mono-ADP-ribose moiety from PARP1 is compromised, resulting in impaired PARP1 transferase activity, associated with defective DNA repair and increased sensitivity to DNA damage. DNA repair deficiency, accompanied by chromosome segregation errors and centrosome amplification, cumulates in CIN contributing to intra-tumor genetic heterogeneity and cancer progression.

Our data suggest a model in which *MACROD2* haploinsufficiency promotes deficient DNA repair and CIN in cancer at least in part through the repression of PARP1 activity (Figure 1). While our data implicate defective HR-mediated DSB repair as a result of PARP1 loss, other PARP1-mediated DNA repair functions such as base excision repair (BER) and non-homologous end joining (NHEJ) pathways may play important roles and remain to be investigated [3]. PARP1 also has central roles in mitosis including centrosome function, HR-dependent repair and restart of stalled replication forks, and mitotic progression.

Functions other than impairment of PARP1 may contribute to the pro-tumorigenic impact of *MACROD2* loss. A previous study identified MACROD2 mono-ADP ribosylhydrolase activity as a regulator of GSK3β function in WNT signaling [9], dysregulation of which is a major driver in multiple malignancies including CRC. However, we found no supporting evidence for MACROD2 regulation of GSK3β function in the context of *APC* or *CTNNB1* mutated CRC cells. Other yet unidentified targets of MACROD2 mono-ADP-ribosylhydrolase activity may be involved. Moreover, MACROD2 has a recognized O-acetyl-ADP-ribose (OAADPr) deacetylase activity that may contribute to its tumor suppressor role.

The use of PARP1 inhibitors in the treatment of HR repair deficient cancers exemplifies the therapeutic paradigm of synthetic lethality in cancer [6]. Characterization of the clinical correlates of *MACROD2* deletions such the predictive value in the setting of current treatments will require retrospective analyses of respective clinical trials. Whether *MACROD2* haploinsufficiency constitutes a vulnerability in tumors that can be specifically exploited using targeted therapeutics is a potential avenue to explore.

Genomic instability is a hallmark of cancer, and CIN is a marker of poor prognosis in multiple cancer types [10]. Our data highlights *MACROD2* as a haploinsufficient caretaker tumor suppressor gene with a pivotal role in the maintenance of genome integrity. While our work focused on CRC, the common focal deletions of *MACROD2* found in stomach, esophagus, cervix, uterus, lung, liver and thyroid cancers suggest a wider role in other human malignancies.
